# libChEBI: an API for accessing the ChEBI database

**DOI:** 10.1186/s13321-016-0123-9

**Published:** 2016-03-01

**Authors:** Neil Swainston, Janna Hastings, Adriano Dekker, Venkatesh Muthukrishnan, John May, Christoph Steinbeck, Pedro Mendes

**Affiliations:** Manchester Centre for Synthetic Biology of Fine and Specialty Chemicals (SYNBIOCHEM), Manchester Institute of Biotechnology, University of Manchester, Manchester, M1 7DN UK; European Bioinformatics Institute, Hinxton, Cambridge, CB10 1SD UK; NextMove Software Ltd., Innovation Centre, Science Park, Milton Road, Cambridge, CB4 0EY UK; School of Computer Science, University of Manchester, Manchester, M13 9PL UK; Center for Quantitative Medicine, UConn Health, Farmington, CT 06030 USA

**Keywords:** Cheminformatics, Database, API, Library, Java, Python, MATLAB, ChEBI

## Abstract

**Background:**

ChEBI is a database and ontology of chemical entities of biological interest. It is widely used as a source of identifiers to facilitate unambiguous reference to chemical entities within biological models, databases, ontologies and literature. ChEBI contains a wealth of chemical data, covering over 46,500 distinct chemical entities, and related data such as chemical formula, charge, molecular mass, structure, synonyms and links to external databases. Furthermore, ChEBI is an ontology, and thus provides meaningful links between chemical entities. Unlike many other resources, ChEBI is fully human-curated, providing a reliable, non-redundant collection of chemical entities and related data. While ChEBI is supported by a web service for programmatic access and a number of download files, it does not have an API library to facilitate the use of ChEBI and its data in cheminformatics software.

**Results:**

To provide this missing functionality, libChEBI, a comprehensive API library for accessing ChEBI data, is introduced. libChEBI is available in Java, Python and MATLAB versions from http://github.com/libChEBI, and provides full programmatic access to all data held within the ChEBI database through a simple and documented API. libChEBI is reliant upon the (automated) download and regular update of flat files that are held locally. As such, libChEBI can be embedded in both on- and off-line software applications.

**Conclusions:**

libChEBI allows better support of ChEBI and its data in the development of new cheminformatics software. Covering three key programming languages, it allows for the entirety of the ChEBI database to be accessed easily and quickly through a simple API. All code is open access and freely available.

**Electronic supplementary material:**

The online version of this article (doi:10.1186/s13321-016-0123-9) contains supplementary material, which is available to authorized users.

## Background

ChEBI is a database and ontology of chemical entities of biological interest [1–3]. With a focus on small molecules, it contains names, chemical structures, synonyms, database cross-references, links to relevant literature, and classifications based on structural features and biological activity. ChEBI has been used as a resource for identifiers for the systematic annotation of chemicals in life science contexts, for example in metabolic models [4–6] and protein [7] and interaction databases [8]. It has also been used as a dictionary of names for chemical text mining [9] and as a source of semantic types for the growing chemical Semantic Web [10, 11].

ChEBI is made available via several access routes. Firstly, it is supported by a website with complex searching and browsing functionality (http://www.ebi.ac.uk/chebi/). Secondly, the data are available for download in full in several different download formats including relational database table data, flat files, the cheminformatics SDfile (structure-data file) format, and ontology formats OBO and OWL. Finally, there is a SOAP-based web service with several access methods that allow search and retrieval of any of the ChEBI content. However, for applications which make a heavy use of ChEBI content, the iterative search-and-retrieve strategy offered by the ChEBI web service may yield insufficient performance, while in order to implement applications which harness ChEBI’s content from many of the different download formats, it is necessary to become familiar with the ChEBI data model. ChEBI is extensively human-curated and, as such, duplicate entries are merged, ensuring that the database contains no redundant entries. Deprecated entries *are* retained but linked to a parent entry, which maintains integrity of the resource and avoids dropped ids and broken links. Due to this added layer of (necessary) complexity, it is inefficient for individual programming efforts to address this issue of id mapping and deprecated entries in repeated independent efforts. libChEBI hides this from the user, ensuring seamless access to all data within the repository.

To facilitate the integration of ChEBI into new and existing software tools, libChEBI, a shared, freely available application programming interface (API) library has been developed. This simple API hides complexity and intricacies of the ChEBI data model, providing a simple interface for accessing ChEBI data. libChEBI has been developed in a generic fashion and will be applicable to any software developers who use (bio)chemical data.

## Implementation

**Fig. 1:**
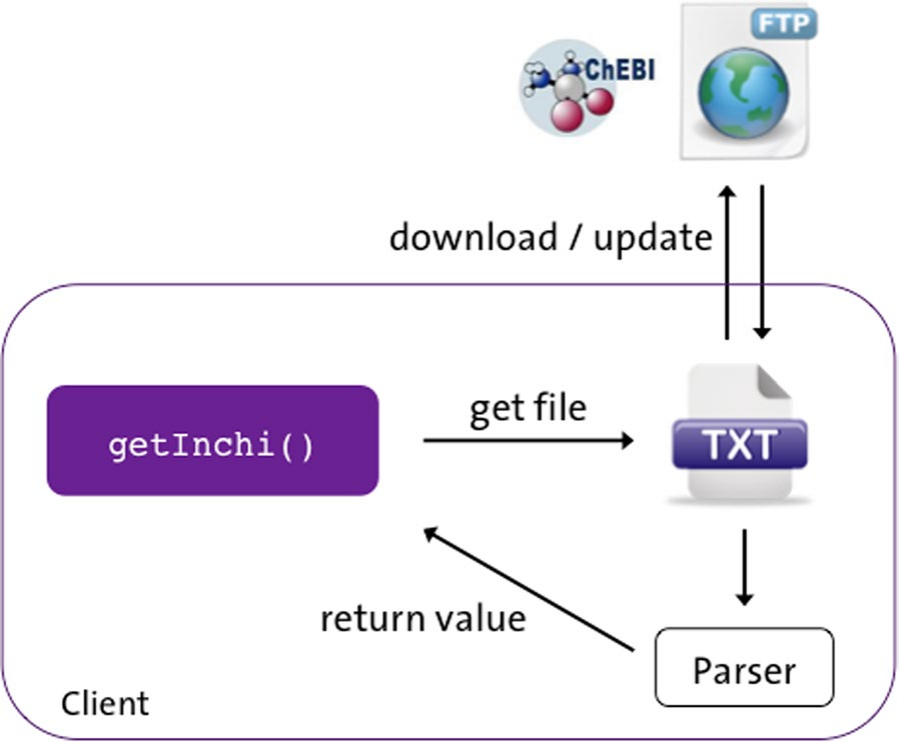
Basic schema of the libChEBI architecture. Upon calling a libChEBI method (e.g. getInchi()), a check is performed to determine whether the current flat text file is available locally. If not, the file is retrieved from the ChEBI FTP site. The flat file is then parsed, and the requested value returned

**Fig. 2:**
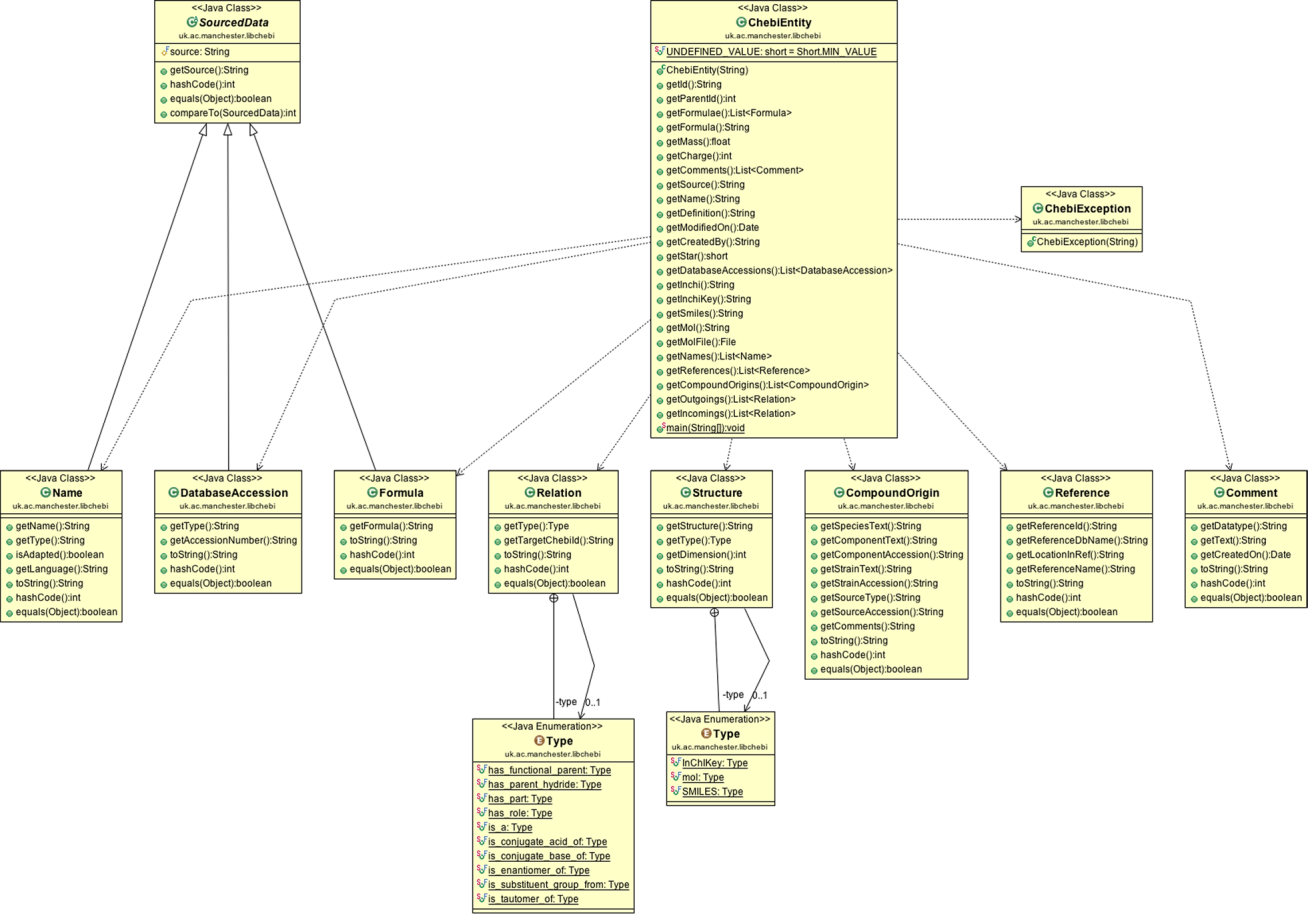
UML class diagram of libChEBIj. All public and protected classes, fields and methods are shown. The Python version, libChEBIpy, follows the same class structure, and these classes are accessible in MATLAB through use of libChEBIm

libChEBI provides a simple interface to the contents of the ChEBI database, built on top of the existing flat file download facility. Flat files are downloaded, unpacked and parsed as required, providing a simple API that is described below. As the flat files are updated on a monthly basis, libChEBI ensures that the most up-to-date version is automatically downloaded. This is the only online requirement of the library, and as such, once the flat files are downloaded, libChEBI can be used offline without any requirement for a connection to the ChEBI database (see Fig. 1). libChEBI provides access to the entire contents of the ChEBI database while removing the need for the user to be familiar with the ChEBI flat file format, or the internal secondary identifier mapping system. Regarding memory issues, the current size (January 2016 release) of all of the unzipped flat files that are parsed is 1.2 GB. However, only a subset of these files (up to 66 MB) is held in memory at any time. Files related to structures and references are not held in memory, as these clearly would cause an excessive memory burden. The library is accessible through Java, Python and MATLAB APIs, which are described in more detail elsewhere (see Fig. 2; Additional file 1: libChEBI API), with examples of use provided below.

## Results and discussion

### Java

The Java public interface consists of a number of classes, of which uk.ac.manchester.ChebiEntity is the primary entry point. The ChebiEntity constructor takes a String, representing the ChEBI id, as a parameter. ChebiEntity then provides a number of methods, providing access to the properties of the ChEBI entity. Example code, illustrating the retrieval of names synonyms for d-glucose, is shown in Box 1.

**Box 1:**
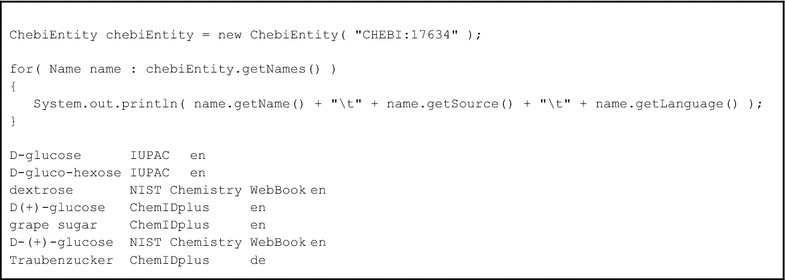
Example libChEBI Java code, illustrating the instantiation of a ChebiEntity, a call to the getNames() method, access of the returned Names objects, and an example of its resulting output

### Python

Like Java, Python is supported by a similar interface with libchebi.ChebiEntity being the primary entry point. Example code is given in Box 2.

**Box 2:**
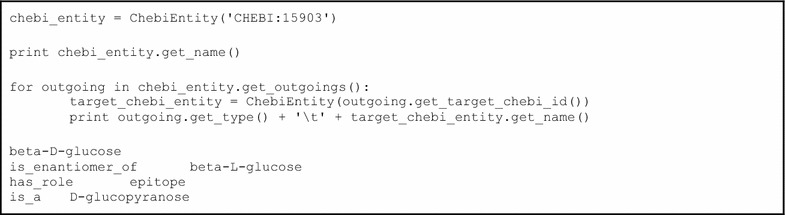
Example libChEBI Python code, showing the instantiation of a ChebiEntity, a call to get_name(), get_outgoings() and the calling of a number of methods of the returned Relation objects

### Matlab

MATLAB support is provided by exploiting the existing facility for bringing Java classes into the MATLAB Workspace. A simple wrapper method, getChebiEntity(id) is provided, which returns a Java uk.ac.manchester.ChebiEntity object. All methods of this object, such as getName(), are then callable from the MATLAB Workspace (see Box 3).

**Box 3:**
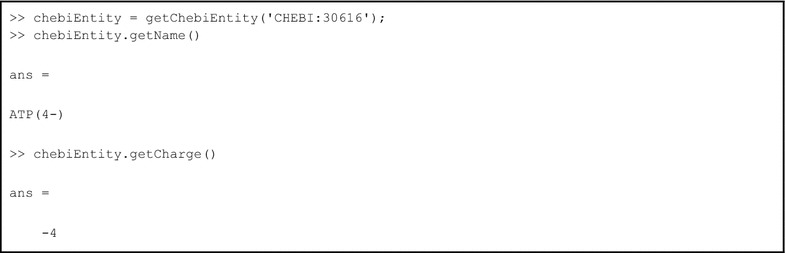
Example libChEBI MATLAB code, illustrating the instantiation of a ChebiEntity, and calls to the getName() and getCharge() methods

### Software application areas utilising ChEBI

In recent years, ChEBI has become increasingly utilised by the systems biology community as a repository of persistent, unambiguous identifiers with which to semantically annotate models. Standardisation of the syntax of systems biology models was addressed with the introduction of the Systems Biology Markup Language (SBML) format over 10 years ago [12]. However, it was recognised that the semantics embedded within these models were non-standardised, with most models containing ambiguous metabolite names and identifiers. Such ambiguity made the interpretation and comparison of such models difficult [13], and their automated parameterisation with experimental data impossible [14, 15]. This issue was partially solved with the introduction of the Minimum Information Requested In the Annotation of Models (MIRIAM) guidelines [16], which provided a facility for annotating model terms with standardised identifiers, such as those provided by ChEBI. Amongst the first large-scale projects to apply these guidelines was that of the Yeast Consensus Model [17, 18], an international collaborative effort to develop a consensus metabolic reconstruction of *Saccharomyces cerevisiae*. This was followed by a similar effort for human metabolism [19, 20], resulting in comprehensive representations of cellular metabolism in which most cellular components are unambiguously identified, a majority of which with ChEBI identifiers.

The use of semantic annotations within models goes beyond just acting as a means of unambiguously identifying components. By providing identifiers linking to publicly available databases, the *content* of these databases can be accessed and used in model refinement, checking and expansion. For example, annotating a model with ChEBI identifiers allows chemical formulae, charge and structural information to be accessed automatically [21]. Such data can then be exploited in model building and checking pipelines such as the SuBliMinaL Toolbox [22], which include automated methods for metabolite charge state determination, reaction balancing and model merging. Application of these methods has led to the automated generation of genome-scale metabolic models of cellular metabolism from over 2000 species [23]. Keeping these models up to date requires automated access to the latest version of ChEBI, which until now required the development and maintenance of custom scripts by each development group, however, such automation is now seamlessly handled through libChEBI.

Although conceived primarily in reference to the requirements within systems biology, libChEBI has been designed in a generic fashion allowing applicability to a range of software applications that utilise chemical data. For example, as the number of annotated metabolites grows, ChEBI is increasingly being used as a reference for metabolite identification and analysis pipelines in metabolomics experiments [24, 25]. Such pipelines currently rely on custom scripts harnessing the SOAP web service, but will now be facilitated. Similarly, within the drug discovery pipeline ChEBI has been used as one of several systems within which chemicals can be classified or grouped in order for patterns to be evaluated in large-scale high-throughput data [26]. As secrecy is important in the drug discovery context, use of the downloadable files from ChEBI is preferred in this context rather than web service queries. However, use of the download files suffers from the complexity of the underlying data model as described above, thus, provision of a targeted library will ease adoption. The reliable human curation and extensive collection of chemical synonyms that are present in the database have resulted in ChEBI becoming a source in text mining applications [27]. ChEBI is also used programmatically within the Bioclipse software platform [28] in diverse contexts including cheminformatics and chemical toxicology. libChEBI has been designed to support both this diverse range of applications and the development of future applications that exploit the contents of the ChEBI database.

## Conclusions

libChEBI is introduced to provide simple programmatic access to the contents of the ChEBI database, and has been designed specifically for developers who wish to incorporate ChEBI data into their software. Future developments may include the support of additional programming languages and implementation of a search facility. However, as a community resource, the direction in which libChEBI develops will be determined by requests from the user community, and as such feedback on this resource is welcomed and encouraged.

## Availability and requirements

- Project name: libChEBI
- Project home page: https://github.com/libChEBI
- Operating system(s): Platform independent
- Programming language: Java, Python, MATLAB
- Other requirements: Java 1.7 or higher, Python 2.7 or higher, MATLAB 2013a or higher
- License: MIT.

## Additional file

10.1186/s13321-016-0123-9 libChEBI API describes the API for each of the supported languages.
