# Supplementary material for: libChEBI: an API for accessing the ChEBI database
Source: J Cheminform. 2016 Mar 1;8:11. doi: 10.1186/s13321-016-0123-9 (PMC4772646; doi:10.1186/s13321-016-0123-9)
Supplement: Supplementary file 1 — 10.1186/s13321-016-0123-9 libChEBI API describes the API for each of the supported languages. [file 13321_2016_123_MOESM1_ESM.pdf]

Additional file 1: libChEBI API

Installation

Java

libChEBIj can be installed through Maven. Add the following dependency to the `pom.xml` file:

```
<dependency>
    <groupId>uk.co.synbiochem</groupId>
    <artifactId>libChEBIj</artifactId>
    <version>1.0.0</version>
</dependency>
```

Additionally, source code is freely available at https://github.com/libChEBI/libChEBIj.

Python

libChEBIpy is installable using pip through the following command:

```
pip install libChEBIpy
```

As an alternative, the source code can be accessed from https://github.com/libChEBI/libChEBIpy.

MATLAB

Source code is available from https://github.com/libChEBI/libChEBIm. Download to a local directory and add the directory to the MATLAB path.

API

The public Java API, libChEBIj, consists of the class `ChebiEntity`, and a number of subclasses, which wraps the flat-file access and parsing, exposing the following interface. Unless otherwise stated, the Java package name is assumed to be `uk.ac.manchester.libchebi`. The Python API, libChEBIpy, follows a similar approach, and with methods having comparable names but following Python convention. For example, the Python equivalent of the Java method `getParentId()` is `get_parent_id()`. libChEBIpy is defined in the module `libchebi`. In the case of MATLAB access, the API exploits MATLAB support of bringing Java classes into its workspace. As a consequence, its API matches that of the Java API.

ChebiEntity

`ChebiEntity(String chebiId)`

Constructor, taking the ChEBI id as parameter.

`String getId()`

Returns the ChEBI id of the ChEBI entity.

String getParentId()

Some ChEBI entities have subsequently been found to be duplicates. If a given entity is a duplicate, it will have a parent id that maps to a parent entity that is considered the primary id for a given set of duplicates. libChEBI manages this mapping of ids to parent ids internally, so a user of libChEBI does not need to be concerned with this feature of the ChEBI data model. However, this method is made available for completeness and can be used to determine if a given id corresponds to a primary or secondary entry: in the former case, `getParentId()` will return `null`.

String getName()

The name for an entity recommended for use by the biological community. In general ChEBI has retained traditional names but these may have been modified to enhance clarity, avoid ambiguity and follow more closely current IUPAC recommendations on chemical nomenclature.

List<Name> getNames()

Returns all alternative names for an entity that either have been used in EBI or external sources or have been devised by the curators based on recommendations of IUPAC, NC-IUBMB or their associated bodies. Returns a `List` of `Name` objects, with the `Name` class being described below.

String getDefinition()

A short verbal definition is included in some entries (and for all new entries annotated after June 2009).

List<Comment> getComments()

A free-text comment may be added to some terms especially in cases where confusing terminology has been historically used. A comment may relate to a single term or to the entry as a whole. Comments are returned as a `List` of `Comment` objects, which are described below.

String getSource()

Returns the source of the ChEBI entity. Examples include ‘ChEBI’, ‘KEGG COMPOUND’ and ‘SUBMITTER’. If the latter, the name of the individual who submitted the entity can be retrieved through `getCreatedBy()`, below.

String getCreatedBy()

If an entry is present by virtue of its having been submitted via the ChEBI Submissions Tool, the name of the submitter is displayed here (unless the submitter has elected to remain anonymous).

Date getModifiedOn()

Indicating the date that the entity was last modified by an annotator.

short getStar()

Entries that have been manually annotated by the ChEBI team are indicated by the presence of a '3-star' symbol. This is shown on the main display screen for an entity and on the search results page. An absence of a '3-star' symbol indicates that a third party has manually annotated the entity, or

(occasionally) that it has been marked as deleted or obsolete.

String getFormula()

Where possible, formulae are assigned for entities and groups. For compounds consisting of discrete molecules, this is generally the molecular formula, a formula according with the relative molecular mass (or the structure). To facilitate searching and downloading of data from external sources, the use of subscripts to indicate multipliers is avoided.

The following conventions regarding ChEBI formulae are followed:

- Unless immediately following a dot '.' any numeral refers to the preceding element in the formula. Example: H2O really means there are two hydrogen atoms and one oxygen atom.
- The dot '.' convention is used when dividing a formula into parts. Any numeral following a dot refers to all the elements within that part of the formula that follow it. Example: C2H3O2.Na.3H2O (CHEBI:32138) really means that after C2H3O2 there is one sodium (Na), six hydrogen and three oxygen atoms.
- Parentheses are used within ChEBI formulae to mean multiplication of elements.
- The 'n' convention is used to show an unknown quantity by which a formula is multiplied. For example: (C12H20O11)n from CHEBI:15443 really means that a C12H20O11 unit is multiplied by an unknown quantity.
- A comma can be used to indicate that there is one or more of the elements divided by the comma but that the exact stoichiometry can vary. For instance, actinolite is a mineral with the chemical formula Ca2(Mg,Fe)5Si8O22(OH)2, which means that it could be anything in the continuous series between Ca2Mg5Si8O22(OH)2 and Ca2Fe5Si8O22(OH)2.

List<Formula> getFormulae()

As `getFormula()`, but will return all representations of chemical formula for a given entity. Some ChEBI entities have conflicting formulae from a number of sources. The formulae and sources are accessible through the `List` of `Formula` objects returned (see `Formula`, below).

int getCharge()

The charge is the sum of all the positive and negative charges shown in the structure. For ions the magnitude of the charge is given in arabic numerals preceded by the sign of the charge. For neutral molecules the charge is indicated as a numerical zero. For instance, the charge of 5,10,15,20-tetrakis(1-methylpyridinium-4-yl)porphyrin (CHEBI:37447) is +4; the charge of borate (CHEBI:22908) is -3.

float getMass()

Returns the average mass. Relative molecular, atomic and ionic masses are shown for molecular, atomic and ionic entities respectively. The relative masses are calculated from tables of relative atomic masses (atomic weights) published by IUPAC.

String getInchi()

The InChI is a non-proprietary identifier for chemical substances that can be used in printed and electronic data sources thus enabling easier linking of diverse data compilations. It expresses chemical structures in terms of atomic connectivity, tautomeric state, isotopes, stereochemistry and electronic charge in order to produce a sequence of machine-readable characters unique to the respective molecule. Further information on the InChI is available at http://www.iupac.org/inchi/. A very useful

'Unofficial InChI FAQ' is also accessible at http://wwmm.ch.cam.ac.uk/inchifaq.

String getInchiKey()

The InChIKey is a 25-character hashed version of the full InChI, designed to allow for easy web searches of chemical compounds. InChIKeys consist of 14 characters resulting from a hash of the connectivity information of the InChI, followed by a hyphen, followed by 8 characters resulting from a hash of the remaining layers of the InChI, followed by a single character indicating the version of InChI used, followed by single checksum character. There is a finite, but very small probability of finding two structures with the same InChIKey. However the probability for duplication of only the first block of 14 characters has been estimated as one duplication in 75 databases each containing one billion unique structures; such duplication therefore appears unlikely at present. Further information on the InChIKey is available at http://old.iupac.org/inchi/release102.html.

String getSmiles()

SMILES (Simplified Molecular Input Line Entry System) is a simple but comprehensive chemical line notation, created in 1986 by David Weininger and further extended by Daylight Chemical Information Systems, Inc. SMILES specifically represents a valence model of a molecule and is widely used as a data exchange format. Further information on SMILES is available at http://www.daylight.com/smiles/.

String getMol()

ChEBI stores the two-dimensional or three-dimensional structural diagrams as connection tables in MDL molfile format. One entity can have one or more connection tables.

File getMolFile()

As `getMol()`, above, but with the molfile data returned in a temporary `File` object.

List<DatabaseAccession> getDatabaseAccessions()

Direct links to the entries for an entity in the databases cited. Essentially a list of cross references to entries in external databases, returned as a `List` of `DatabaseAccession` objects, which are described more fully below.

List<Reference> getReferences()

Returns a list of textual references to external resources, covering references to the given ChEBI entity in resources such as BRENDA, UniProt and in patents. A description of the `Reference` object is given below.

List<CompoundOrigin> getCompoundOrigins()

Provides a list of origins for a given ChEBI entity. These are essentially records of taxonomy terms, indicating species in which the chemical entity has been detected. This data is accessible from a `List` of `CompoundOrigins`, described below.

List<Relation> getOutgoings()

ChEBI is an ontology, and as such maintains relations between chemical entities. These are essentially triples of the format subject-predicate-object. Outgoing relations, in which the ChEBI entity is the subject

of the relationship, can be accessed through the `getOutgoings()` method. This returns a `List` of `Relation` objects.

`List<Relation> getIncomings()`

Similar to `getOutgoings()`, `getIncomings()` returns a `List` of `Relation` objects stating relations in which the ChEBI entity is the object of the relationship.

Name

Class to represent a chemical name or synonym. A given ChEBI entity may have many synonymous names.

`String getName()`

The name itself.

`String getType()`

The name type, with examples being 'NAME' (the standard name provided by ChEBI, and returned by the ChebiEntity.getName() method), 'IUPAC NAME' and 'SYNONYM'.

`boolean isAdapted()`

When a change has been made to a name relative to the version used in the source from which the name originates, the adapted flag will be set to true. This ensures transparency with respect to the original source. This is only used for minor changes: if major changes needed to be introduced, the result would be included as a separate name.

`String getLanguage()`

The language of the name, in two-letter country code such as 'en' and 'de'.

String getSource()

The source of the name. Typically takes the value of "ChEBI" or an external data resource such as "KEGG COMPOUND" or "UniProt".

Comment

Free-text comments, primarily curation notes, are provided in the `Comment` class.

`String getDatatype()`

The ChEBI entity data field to which the comment refers. Includes values such as "CompoundName", "DatabaseAccession" and "General".

`String getText()`

The text of the comment.

`Date getCreatedOn()`

The date on which the comment was made.

Formula

ChEBI entities may have a number of associated formulae from different sources. This information is returned in classes of type Formula.

`String getFormula()`

The chemical formula, following the convention as described in `ChebiEntity.getFormula()`, above.

`String getSource()`

The source of the formula. This is typically ‘ChEBI’, as ChEBI curators normally provide formulae. Other values include ‘KEGG COMPOUND’ and ‘SUBMITTER’ for database imports and user submissions, respectively.

DatabaseAccession

The `DatabaseAccession` class describes a cross reference to an entry in an external data resource.

`String getType()`

The type of cross-reference, which typically corresponds to the linked data resource. Values include ‘CAS Registry Number’, ‘Wikipedia accession’ and ‘PubMed citation’.

`String getAccessionNumber()`

The accession number of the entry in the external database.

`String getSource()`

The source of the cross-reference. Includes sources such as ‘KEGG COMPOUND’, ‘MetaCyc’ and ‘Wikipedia’.

Reference

A reference (not a database entry cross reference, see `DatabaseAccession` above) indicating “mentions” of a given ChEBI entity in an external resource.

`String getReferenceId()`

An id of the reference in the external resource. Can be, for example, a UniProt id or a patent id.

`String getReferenceDbName()`

The name of the external resource. Examples include ‘UniProt’, ‘Patent’, ‘ChEMBL’ and ‘Rhea’.

`String getLocationInRef()`

In some cases, a specific field within an external resource entry can be provided. An example of this is with UniProt references, where specific fields in which the reference occurs, such as ‘CC - CATALYTIC ACTIVITY’, can be accessed.

String getReferenceName()

Returns a human-readable name of the reference itself.

CompoundOrigin

`CompoundOrigin` describes the biological origin of the ChEBI entity, including fields such as species and strain, describing the organism in which a given ChEBI entity is present.

String getSpeciesText()

The species name in textual format, such as “Saccharomyces cerevisiae”.

String getSpeciesAccession()

The species accession number, and associated data resource. These are typically references to entries in the NCBI Taxonomy database, and have the format “NCBI:4932”.

String getComponentText()

The biological component, in textual format. An example being “leaf”. This field is often `null`.

String getComponentAccession()

As above, but returned as a database accession and associated data resource. Commonly referencing the Brenda Tissue Ontology, a typical example is “BTO:0000713”.

String getStrainText()

Text field describing the strain.

String getSourceType()

A textual representation of the source of the data. Examples include “PubMed Id” and “DOI”.

String getSourceAccession()

Corresponds with the source type, above, and returns the accession number of the data resource entry from which the origin data was extracted. Examples include PubMed ids or DOIs such as “10.1038/nbt.2488”.

String getComments()

Free text field holding curator comments regarding the `CompoundOrigin`.

Relation

A `Relation` object describes a relationship in the ChEBI ontology.

String getTargetChebiId()

The id of the other ChEBI entity involved in the relationship. If the relationship is outgoing, this corresponds to the object of the subject-predicate-object triple. If incoming, this id refers to the subject.

`Type getType()`

The `Type` of `Relation` describes the predicate. It is an `enum`, and can take the following values: `has_functional_parent`, `has_parent_hydride`, `has_part`, `has_role`, `is_a`, `is_conjugate_acid_of`, `is_conjugate_base_of`, `is_enantiomer_of`, `is_substituent_group_from`, `is_tautomer_of`.
